# Overt Lower Gastrointestinal Bleeding and Pseudotumor: A Rare Presentation of Cytomegalovirus Infection

**DOI:** 10.1155/2017/9732967

**Published:** 2017-10-18

**Authors:** Akanksha Agrawal, Deepanshu Jain, Sameer Siddique

**Affiliations:** ^1^Department of Internal Medicine, Albert Einstein Medical Center, Philadelphia, PA, USA; ^2^Department of Digestive Diseases and Transplantation, Albert Einstein Medical Center, Philadelphia, PA, USA

## Abstract

Cytomegalovirus (CMV) is a ubiquitous organism which can infect multiple organs of the body. In an immunocompromised patient, it can have a myriad of gastrointestinal manifestations. We report a case of recurrent hematochezia and concomitant pseudotumor in an AIDS (acquired immunodeficiency syndrome) patient attributable to CMV infection. A 62-year-old man with a history of AIDS, noncompliant with highly active antiretroviral therapy (HAART), presented with bright red blood per rectum. Index colonoscopy showed presence of multiple ulcers, colonic stenosis, and mass-like appearing lesion. Biopsy confirmed CMV infection and ruled out malignancy. Cessation of dual antiplatelet therapy and compliance with HAART lead to clinical cessation of bleeding and endoscopic healing of ulcers with complete resolution of colon mass on follow-up colonoscopy.

## 1. Introduction

Cytomegalovirus (CMV) is a ubiquitous organism and can infect the lung, eye, gastrointestinal tract, brain, etc., with most being subclinical and asymptomatic. In an immunocompromised patient, CMV infection of colon can present in a variety of ways: ulcers, proctocolitis, pseudotumors, pseudomembranes, appendicitis, perforation, pneumatosis intestinalis, or toxic megacolon [1]. We report a case of CMV causing recurrent, significant hematochezia and concomitant presence of a discrete mass in the colon of an AIDS (acquired immunodeficiency syndrome) patient.

## 2. Case

**Figure 1:**
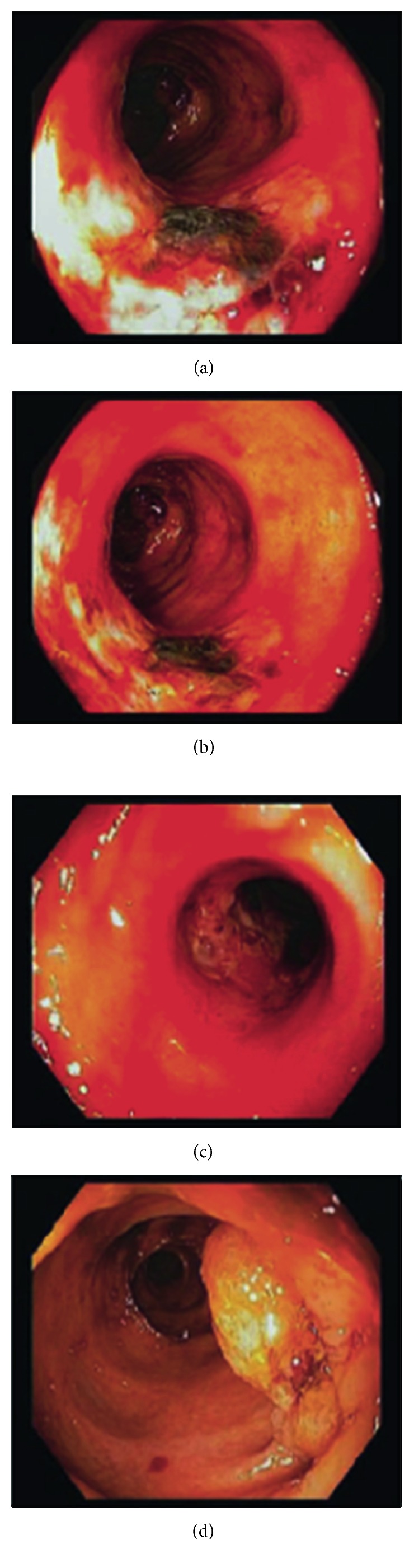
(a), (b), and (c) represent two stenosed ulcerative colon segments, and (d) represents pseudotumor in a stenosed colon segment at the index colonoscopy.

**Figure 2:**
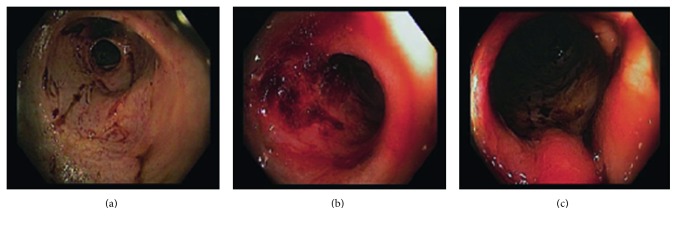
Stenosed colon segment with minimal erythema at second colonoscopy.

A 62-year-old man with a history of end stage renal disease on hemodialysis, AIDS being noncompliant with highly active antiretroviral therapy (HAART), coronary artery disease on aspirin and clopidogrel, compensated alcoholic cirrhosis, stroke, and hypertension presented with bright red blood per rectum for two weeks. He denied presence of nausea, vomiting, abdominal pain, diarrhea, or significant weight loss. Vitals were stable, and lab work was remarkable for pancytopenia (hemoglobin 6.3 g/dL, platelet 70/mcL, and white blood cell 2.0/mcL). Liver function tests and coagulation markers were in normal range, and CD4 count was 212/mm^3^. Colonoscopy showed two stenosed ulcerated short segments in transverse colon with active oozing (requiring epinephrine injection therapy) and normal looking mucosa in between the lesions (Figures 1()–1()). Another stenosed segment with mass-like appearance was also noted in the transverse colon giving the macroscopic appearance of a mass (Figure 1(d)). Biopsy from ulcer and mass showed presence of fibrinopurulent debris with granulation tissue. No neoplastic cells or infectious etiology was identified. He underwent an EGD which showed similar lesions but with no overt bleeding or stigmata of recent hemorrhage. The bleeding resolved, and the patient was discharged with emphasis on compliance with HAART and with instructions to attend outpatient GI clinic, suspecting an infectious etiology. Three weeks after his index admission, he was readmitted for recurrent symptoms of acute onset bright red blood per rectum and drop in hemoglobin by 5 g/dl. Repeat colonoscopy revealed healing stenosed segments at the same location with minimal erythema (Figures 2()–2()) with the overall impression being of improving colitis. Biopsy from the lesions showed rare CMV-positive cells in a background of granulation tissue. The previously seen mass-like lesion had improved to an ulcerated area on this repeat colonoscopy. The initial tumor-like appearance but lack of malignant cells on histopathology and its resolution on follow-up colonoscopy with HAART confirmed our initial suspicion of a pseudotumor. Due to his recurrent GI bleeding with background thrombocytopenia, a discussion with the cardiologists resulted in a coronary angiography showing absence of significant occlusive disease and consequent cessation of dual antiplatelet therapy.

**Figure 3:**
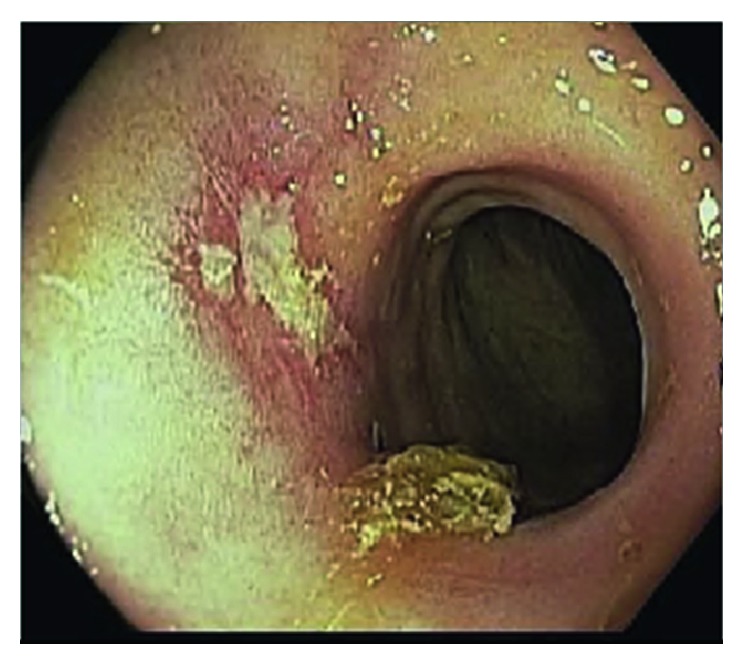
Stenosed colon segment with isolated healing erosion at 3-month colonoscopy.

Objective improvement in the colonic lesions based on serial colonoscopies with improved compliance with HAART therapy precluded the need for CMV-directed therapy. At the time of discharge, the patient was recounselled on the need of compliance with HAART. A 3-month follow-up colonoscopy showed continued healing at the stenosis site and absence of pseudotumor (Figure 3()).

## 3. Discussion

With ever-increasing use of immunosuppressive/immunomodulatory medications and cancer chemotherapy, coupled with a progressively aging population, and resurgent human immunodeficiency virus infections, CMV is being recognized as a pathogen that can present in myriad manifestations [2]. Gastrointestinal CMV is an erosive or ulcerative disease, which can occur at any location in the gastrointestinal tract from mouth to rectum, colon being the most common [2]. The pathogenesis involves infection of the columnar epithelial cell, endothelial cell, myocyte, and fibroblast causing tissue destruction and ulceration. The endoscopic finding can vary from normal appearing mucosa to diffuse erythema, nodules, pseudotumor, erosions, and ulcerations [3]. Significant gastrointestinal bleeding from CMV infection has never been reported in presence of concomitant pseudotumor.

Diagnosis of gastrointestinal CMV is made by a mucosal biopsy showing presence of CMV either histopathologically or by using other techniques. CMV infection can sometimes be missed on gastrointestinal biopsy, like in our case at the first colonoscopy. This is likely attributed to location of the virus in the deep tissue. Hence, biopsy should be performed deep enough to gather endothelial cells and fibroblasts in the lamina propria [4]. Our second biopsy proved the presence of CMV using immunohistochemistry.

In patients with AIDS, Kaposi's sarcoma and lymphomas are the most frequent malignancies of the gastrointestinal tract, accounting for nearly 95% of all identified malignancies [5, 6]. The rarer causes include infectious causes like tuberculosis, toxoplasmosis, histoplasmosis, and CMV infection. The macroscopic appearance of a mass on colonoscopy, negative biopsy for malignancy, and prompt resolution of mass with HAART proved that our patient had pseudotumor due to CMV infection.

The pathogenesis of CMV pseudotumor remains unclear. It is hypothesized that thickening of bowel wall is histologically characterized by submucosal edema, granulation tissue, and fibrosis. Due to such exuberant formation of granulation tissue and inflammation in response to CMV, there occurs formation of CMV-induced inflammatory mass or pseudotumor [7]. The reason why sometimes CMV would have ulcerative process and rarely lead to such proliferative process is not known.

Cellular immunity plays a key role in controlling CMV in healthy and immunocompromised individuals. In patients with persistent immune deficiency, the disease can progress to fatal outcomes like perforation and death. Deayton et al. showed a rapid decline in CMV DNA levels in the blood after initiation of HAART in HIV patients [8]. They further concluded that HAART effectively inhibits CMV replication to as high as 85%, thereby proving effective antiviral activity against CMV infection. Another study by Weinberg et al. showed similar findings [9]. Other than HAART, there are three systemic drugs approved for the treatment of CMV: ganciclovir or its prodrug valganciclovir, foscarnet, and cidofovir. Our patient showed improvement in the lesions with HAART therapy on serial colonoscopies. Hence, it was decided to not use a specific agent but continue the emphasis on compliance with HAART.

## 4. Conclusion

Our case highlights several important clinical aspects of diagnosis and management of CMV infection in an immunocompromised patient. An astute physician should always keep in mind the atypical presentations of CMV, importance of proper biopsy technique, and the use of HAART therapy alone as a treatment for colonic manifestations of CMV in AIDS patients.
